# Imidacloprid Induces Lysosomal Dysfunction and Cell Death in Human Astrocytes and Fibroblasts—Environmental Implication of a Clinical Case Report

**DOI:** 10.3390/cells12242772

**Published:** 2023-12-05

**Authors:** Ida Eriksson, Liam J. Ward, Linda Vainikka, Nargis Sultana, Per Leanderson, Ulf Flodin, Wei Li, Xi-Ming Yuan

**Affiliations:** 1 Occupational and Environmental Medicine, Department of Health, Medicine and Caring Sciences, Linköping University, 581 85 Linköping, Sweden; per.leandersson@regionostergotland.se (P.L.); ulf.flodin@regionostergotland.se (U.F.); ximing.yuan@liu.se (X.-M.Y.); 2 Experimental Pathology, Department of Biomedical and Clinical Sciences, Linköping University, 581 85 Linköping, Sweden; ida.eriksson@liu.se (I.E.); linda.vainikka@liu.se (L.V.); 3 Obstetrics and Gynaecology, Department of Biomedical and Clinical Sciences, Linköping University, 581 85 Linköping, Sweden; wei.li@liu.se (W.L.); 4 Laboratory Medicine, Linköping University Hospital, 581 85 Linköping, Sweden; nargis.sultana@regionostergotland.se (N.S.); 5 Department of Forensic Genetics and Forensic Toxicology, National Board of Forensic Medicine, 587 85 Linköping, Sweden; liam.ward@rmv.se (L.J.W.)

**Keywords:** autophagy, apoptosis, central nervous system, glial cells, lysosomes, necrosis, neonicotinoid insecticide, occupational exposure, oxidative stress

## Abstract

Imidacloprid (IMI), a neonicotinoid insecticide, has potential cytotoxic and genotoxic effects on human and experimental models, respectively. While being an emerging environmental contaminant, occupational exposure and related cellular mechanisms are unknown. Herein, we were motivated by a specific patient case where occupational exposure to an IMI-containing plant protection product was associated with the diagnosis of Bell’s palsy. The aim was to investigate the toxic effects and cellular mechanisms of IMI exposure on glial cells (D384 human astrocytes) and on human fibroblasts (AG01518). IMI-treated astrocytes showed a reduction in cell number and dose-dependent cytotoxicity at 24 h. Lower doses of IMI induced reactive oxygen species (ROS) and lysosomal membrane permeabilisation (LMP), causing apoptosis and autophagic dysfunction, while high doses caused significant necrotic cell death. Using normal fibroblasts, we found that IMI-induced autophagic dysfunction and lysosomal damage, activated lysophagy, and resulted in a compensatory increase in lysosomes. In conclusion, the observed IMI-induced effects on human glial cells and fibroblasts provide a possible link between IMI cytotoxicity and neurological complications observed clinically in the patient exposed to this neonicotinoid insecticide.

## 1. Introduction

Imidacloprid (IMI) is a synthetic neonicotinoid insecticide that has been used worldwide for plant protection and agricultural purposes since 1991. The method of action for IMI involves the blockade of the nicotinergic neuronal pathways in the central nervous system (CNS), leading to an accumulation of acetylcholine and resulting in paralysis and eventual death of an insect [1]. Recently, IMI has been highlighted as an emerging contaminant due to its high usage and toxic effect on a variety of non-target organisms, including humans [2].

In 2018, IMI and two other neonicotinoids, clothianidin and thiamethoxam, were permanently banned by the European Commission for plant protection use in all outdoor cultivations. However, use in indoor greenhouse environments is still permitted if the treated plants remain within the greenhouse for the duration of their life cycle [3]. The ever-increasing use of indoor cultivation within the agricultural industry coincides with IMI being the most-sold insecticide worldwide and the third most sold by weight in Sweden [4]. The increased use of IMI as an insecticide in indoor greenhouse environments has raised concerns about the potential occupational impact on workers. Water monitoring of streams adjacent to commercial greenhouses in southern Sweden identified IMI as the most common pesticide [4], in some cases with IMI in surface water downstream of greenhouses being twice the order of magnitude greater than those found within high-intensity agricultural areas [5]. Moreover, new data have evidenced that exposure to IMI, and other neonicotinoids, in seabirds needs future investigations into the extent of neonicotinoid contamination in non-agricultural ecosystems [6]. 

The toxicity of IMI has been assessed in vitro in various human cell lines, including lymphocytes [7,8], WPM-Y.1 prostate epithelial cells [9], A549 pulmonary epithelial cells [10], and HepG2 hepatocellular carcinoma cells [11]. Furthermore, the toxicity of IMI has been assessed in SH-SY5Y neuroblastoma cells as an established model of the effects of IMI on nicotinic acetylcholine receptor (nAChR) signalling [10,11,12]. IMI exposure induced apoptotic and oxidative stress effects in WPM-Y.1 cells [9] and arrested cell growth at 50% inhibitory concentrations (IC50) of 1.8 mM and 1.5 mM in the A59 and SH-SY5Y cell lines after 24 h, respectively [10]. In addition, several recent studies have assessed IMI toxicity in animal models, including zebrafish [13]. Interestingly, all three studies show neurological irregularities in response to IMI treatment [13]. IMI altered gene expressions related to oxidative stress and neurological development in zebrafish embryos [14], induced reactive oxygen species (ROS) production and severe damage to glial cells in Drosophila [15], and impaired neurogenesis and altered glial profiles in mouse neonates [13]. Of note, both neuronal and non-neuronal CNS cell types have been implicated in IMI toxicity, with SH-SY5Y neuroblastoma cells being used to assess IMI toxicity in vitro [10,11,12] and animal models highlighting alterations in glial cells with impaired neurogenesis via ROS-triggered neurological and metabolic impairments [13,15].

The environmental concern of neonicotinoid contamination is evident; however, investigations on the potential occupational impact on workers are limited. In this study, we were motivated by a specific patient case where the patient had worked, unprotected, with a plant protection product containing IMI. The patient developed muscle weakness in the forehead and corner of the mouth and was diagnosed with Bell’s palsy (facial muscle weakness or paralysis). Thus, we aimed to investigate the possible toxic effects and cellular mechanisms of IMI exposure on glial cells using the human astrocytoma cell line (D384) and human fibroblasts (AG01518).

## 2. Materials and Methods

### 2.1. Case Description

A patient, from September 2011, has been employed as a machine operator at a forestry contractor company. The patient worked planting spruce seedlings in the forest and was exposed to plant protection products containing imidacloprid (70%; Merit Forest WG, Leverkusen, Germany) and copper hydroxide (323 g/L; Spin Out 300) for 8 h a day, 5 days a week, year-round for 2 years. In total, the patient worked between 40 and 50 h per week without the use of personal protective equipment, such as clothing or a face mask. Most of this job was completed in the spring season. The patient did not know about the potential hazard of substances in the plant protection products and only wore ordinary gloves that had not been changed for several months. The patient’s work clothes had often become wet from the products, and the patient changed and washed clothes at home. With assistance from a worker’s union, the patient received information, concerning the active substances in the plant protection products—including imidacloprid in Merit Forest WG, and copper hydroxide in Spin Out 300. 

### 2.2. Cell Cultures and Experimental Conditions

The human D384 astrocytoma cell line (ATCC, Gaithersburg, MD, USA) and human AG01518 fibroblasts were grown in Dulbecco’s modified Eagle’s minimal medium (GIBCO, Thermo Fisher Scientific, Fresno, CA, USA) with 10% (*v*/*v*) heat-inactivated foetal calf serum, with ≤5 EU/mL content of endotoxin (GIBCO), and 1% penicillinstreptomycin (MP Biomedicals, France). The D384 cells were sub-cultivated twice a week and used for experiments within 24 h. All experiments were performed in 35 mm culture dishes on coverslips containing approx. 3 × 10^5^ cells, except for the DNA quantification assay, where cells were cultured on 96-well plates.

Fibroblasts (passages 13–23) were sub-cultivated once a week, trypsinized, and seeded at a density of 10,000 cells/cm^2^. This allowed a confluency of 80% at the time of IMI exposure. Bafilomycin A1 (Sigma-Aldrich, St. Louis, MO, USA) was used at a concentration of 25 nM and 3-methyladenine (3-MA, Sigma-Aldrich) at a concentration of 5 mM. Both inhibitors were added together with IMI and were present during the entire time of IMI exposure.

IMI (Merit Forest WG; Bayer Environmental Science, Leverkusen, Germany) was dissolved in dimethyl sulfoxide (DMSO, Sigma-Aldrich, MO, USA) to a stock concentration of 400 mM. Test solutions of IMI were prepared freshly just before application by diluting the stock solution in culture media to a concentration of 4 mM. The final concentration of DMSO in the cell suspension did not exceed 0.2%, with a final concentration of IMI at 0.8 mM.

It is difficult to determine the concentration of IMI exposure for an in vitro model that can imitate the related occupational exposure. Additionally, there are no previous studies on human astrocyte cells and IMI exposure. However, several in vitro studies have quantified responses of human nAChRs and toxicity after IMI exposure at concentrations ranging from 0.1 to 4 mM [11,12,16,17]. Thus, in the experiments, the cells were exposed to various concentrations of IMI (0.02–2 mM), or not (controls), for varying periods of time and then used for analyses of cell viability, lysosomal membrane permeabilisation (LMP), cellular ROS production, and autophagy protein markers.

### 2.3. Cell Number and DNA Content

Cellular DNA content is closely proportional to cell number. Therefore, changes in nucleic acid content can serve as a sensitive indicator of cell number, as well as cytotoxic events or pathological abnormalities that affect cell proliferation. Hoechst 33342, a non-toxic specific vital stain for DNA, interacts with cell chromatin DNA, resulting in fluorescence [18]. Briefly, cells were grown in 96-well plates with or without treatment conditions. After the treatment period, cells were washed and then further cultured for 48 h under standard culture conditions. For the DNA staining, media were aspirated from the well, followed by washing, SDS cell lysis, and staining with Hoechst dye. Fluorescence was measured with a fluorometer, using 350 nm and 460 nm as excitation and emission wavelengths, respectively.

### 2.4. Apoptosis and Necrosis

Cell viability was assayed morphologically and with the Trypan blue dye (0.05%, 25 °C, 5 min) exclusion test. Cells positive for Trypan blue in the nuclei were considered as necrotic and presented as a percentage of the total number of cells. Viability was also analysed using the MTT (3-[4,5-Dimethylthiazol-2-yl]-2,5-diphenyltetrazolium bromide; Calbiochem, San Diego, CA, USA) reduction assay. Cells were incubated with 0.25 mg/mL MTT for 3 h at 37 °C, after which the formazan product was dissolved in DMSO. Absorbance was analysed at 550 nm using a SPARK 10M Microplate Reader (Tecan, Männedorf, Switzerland).

Apoptotic cells were assayed by detection of phosphatidylserine exposure using confocal microscopy following Annexin V/Propidium iodine (AV/PI) staining (Roche Diagnostics, Mannheim, Germany). Briefly, control and treated cells were washed once with PBS and then stained with AV/PI for 20 min on ice before the percentage of apoptotic cells was analysed.

### 2.5. Reactive Oxygen Species (ROS)

Intracellular ROS were assessed using fluorescence microscopy following dihydroethidium (DHE; Molecular Probes, Eugene, OR, USA) staining. DHE is one of the most widely used fluorogenic probes for the detection of intracellular superoxide and ROS production. Cells were incubated for 15 min at 37 °C with 10 µM DHE and analysed using a Zeiss confocal LSM 700 microscope. 

### 2.6. Lysosomal Membrane Permeabilisation (LMP)

LMP was assessed using the acridine orange (AO) uptake technique, as established previously [19]. Herein, live cells were stained for 15 min with 2 mL AO solution (in D384 cells 5 μg/mL and in fibroblasts 2 µg/mL in complete medium) at 37 °C after each time point of treatment with IMI and analysed using a Zeiss confocal LSM 700 and a Zeiss confocal LSM 800 microscope (Carl Zeiss AB, Stockholm, Sweden). 

### 2.7. Immunocytochemistry of D384 Astrocytes 

Cells grown on coverslips were either treated with or without IMI for 24 h, fixed with 4% (*w*/*v*) formaldehyde for 20 min, and permeabilised with buffer containing 0.1 g saponin and 5% serum in PBS. Primary antibody incubation with rabbit anti-human LC3 (1:200, overnight, 4 °C; Novus Biological, Abingdon, United Kingdom https://www.novusbio.com/products/lc3b-antibody_nb100-2220) or rabbit anti-human p62/SQSTM1 (1:200, overnight, 4 °C; MBL International Corporation, TÄBY, Sweden, https://www.mblintl.com/products/wp-content/uploads/sites/2/2020/08/PI-PM045-Rev.-7-1.pdf) was followed by secondary antibody incubation for 1 h at room temperature with goat anti-rabbit Alexa Fluor 568 (1:200; Invitrogen, Waltham, MA, USA) or Alexa Fluor 488 (1:200; Invitrogen), respectively. All immunostained cells were mounted with DAPI-containing mounting media (Vector Laboratories, Burlington, ON, Canada), and analysed using a Zeiss confocal LSM 700 microscope. Images were taken and processed using Zen Lite 2011 software (Carl Zeiss AB) with a 40× oil-immersion objective. Controls without primary antibodies or with non-immune IgG were used, resulting in consistently negative results. Images were analysed for the presence of LC3+ puncta as previously described [20] and using Image J software (https://imagej.net/ij/) [21].

### 2.8. Immunocytochemistry of AG01518 Fibroblasts 

AG01518 fibroblasts grown on coverslips were exposed to 0.8 mM IMI for 48 h, fixed with 4% (*w*/*v*) formaldehyde for 20 min, and permeabilised with buffer containing 0.1 g saponin and 5% serum in PBS. Specimens were incubated with primary antibody (rabbit anti-human LC3, 1:200, Novus Biological, Sweden; mouse anti-human p62/SQSTM1, 1:400, Proteintech, Rosemont, IL, USA; rabbit anti-human LAMP2a; 1:100, Abcam, Cambridge, UK, or mouse anti-human galectin-3; BD Pharmingen, San Diego, CA, USA) over night at 4 °C, followed by secondary antibody (goat anti-rabbit Alexa Fluor 488 or goat anti-mouse Alexa Fluor 546, 1:400, Invitrogen) for 1 h at room temperature. Cells were mounted in ProLong Diamond Antifade Reagent (Invitrogen) and analysed using a Zeiss confocal LSM 800 microscope. Images were taken and processed using Zen Blue 3.1 software (Carl Zeiss AB) with a 40× oil-immersion objective. Controls without primary antibodies or with non-immune IgG were used, resulting in consistently negative results.

### 2.9. Western Blot

Samples were subjected to Western blot, as described previously [22]. Briefly, 10–25 µg of protein was separated using a 12% Bolt Bis-Tris Plus protein gel (Invitrogen) and then transferred onto a nitrocellulose membrane with an iBlot 2 transfer device (Thermo Fisher Scientific). The membrane was probed with a primary antibody (rabbit anti-human LC3, 1:1000, Novus Biological; mouse anti-human p62/SQSTM, 1:1000, Proteintech; LAMP2, 1:1000, Southern Biotech, Homewood, AL, USA) overnight at 4 °C, followed by an HRP-conjugated goat anti-mouse or goat anti-rabbit secondary antibody (1:3000, Dako, Glostrup, Denmark). Equal loading was verified using HRP-conjugated mouse anti-human glyceraldehyde-3-phosphate dehydrogenase (GAPDH) (1:20 000, Novus Biological). Proteins were visualised using Clarity ECL Substrate (Bio-Rad Laboratories, Hercules, CA, USA) and captured digitally with the Chemidoc XRS system (Bio-Rad Laboratories). Densitometric analysis was performed using Image Lab Software (https://www.bio-rad.com/zh-cn/product/image-lab-software?ID=KRE6P5E8Z) (Bio-Rad Laboratories).

### 2.10. Statistics 

For statistical analyses, a one-way ANOVA followed by a Bonferroni post hoc test for multiple comparisons was performed, while an unpaired *t*-test was used for the comparison of 2 groups. Data are presented as mean ± SD unless otherwise stated, and statistical significance was set at a *p*-value < 0.05. The major statistical conclusions were further confirmed using a nonparametric comparison Kruskal–Wallis test with Dunn’s post hoc test (for the comparison of 3 groups) or the Mann–Whitney U test (for the comparison of 2 groups).

## 3. Results

### 3.1. Health Problems of the Patient 

The patient presented with sudden illness, in March 2012, due to right-sided facial paralysis, associated tinnitus, and slow blinking/inability to close the right eye. Additionally, hearing loss and increased sound sensitivity were experienced in the right ear. The patient developed muscle weakness in the forehead and the corner of the mouth and was diagnosed with Bell’s palsy. The patient also experienced other symptoms such as severe dry mouth, discomfort in the stomach due to gas, and a minor headache. There were no experiences with dizziness or erythema/rash. The patient had contact with the ear, nose, and throat clinic on multiple occasions up until the described symptoms disappeared after a long period of sick leave. The patient had been on 100% sick leave between March 2012 and June 2014, and, by that time, was reluctant to return to work to undertake the same tasks.

### 3.2. IMI Exposure Decreases Cell Proliferation and Induces Cell Death in Human D384 Astrocyte and Human Fibroblast Cells

**Figure 1:**
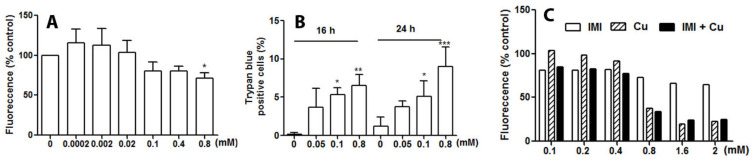
Exposure to IMI decreases DNA synthesis and results in cell death of human astrocytoma cell line D384 cells. (**A**) D384 cells were cultured in 96-well plates with different concentrations of IMI for 24 h. Assays of DNA content in fluorescence signal quantification were performed according to the method described. Data are based on 2–5 independent experiments. * *p* < 0.05 as compared with IMI 0.0002 mM. (**B**) D384 cells on coverslips were stained with Trypan blue and analysed with light microscopy. Data are based on three independent experiments: * *p* < 0.05, ** *p* < 0.01, and *** *p* < 0.001 as compared with control cells. (**C**) Exposure to combined copper with IMI (24 h) results in a similar toxic response as compared with exposure to only copper. Data are based on two independent experiments.

To address the cytotoxicity of IMI towards human glial cells, D384 astrocytes were exposed to various concentrations of IMI for time intervals up to 48 h. IMI exposure at different concentrations resulted in a decrease in DNA content in human astrocytes. The reduction in DNA content was about 20% of controls when the cells were exposed to 0.1–0.4 mM without statistical significance. A significant decrease in DNA content was seen in the cells treated with 0.8 mM IMI, indicating a reduction in the number of cells after the treatments (Figure 1A). IMI exposure was cytotoxic at 16 and 24 h when the cells were exposed to IMI between concentrations of 0.05 and 0.8 mM, and a dose-dependent reduction in cell viability was significant at 0.1–0.8 mM exposure (Figure 1B). 

The case patient experienced mixed occupational exposure to both IMI and copper hydroxide, the active ingredient in Spin Out 300, at a concentration of 323 g/L. Thus, we investigated the synergistic toxic potential of IMI and copper on human astrocytes. In a series of experiments, astrocytes were treated for 24 h using different concentrations of copper (0.1–2 mM), IMI, and a combination of the two compounds (Figure 1C). Representatively, both compounds showed toxicity at a concentration of 0.8 mM, with copper being comparatively more toxic than IMI, and the combined treatment resulted in similar toxic effects as exposure to copper alone (Figure 1C). As no additive synergism was observed with the addition of IMI to copper, no further experiments using the combined treatment were performed.

**Figure 2:**
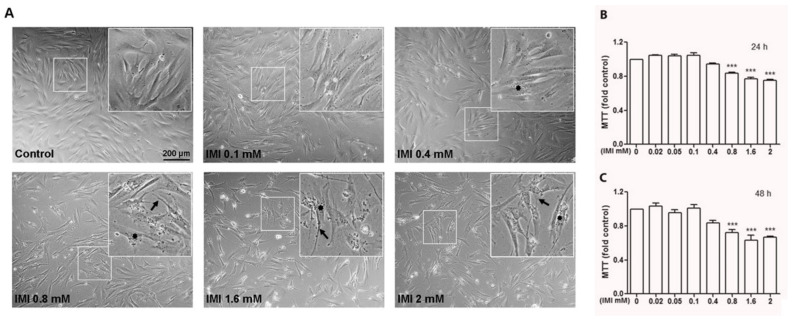
Exposure to IMI results in cellular shrinkage and cell death of human fibroblast cells. (**A**) Human fibroblasts were cultured in 35 mm culture plates with different concentrations of IMI for 48 h (0.02–2 mM). (**A**) Cell morphology was assayed with light microscopy without staining. Arrows indicate cell membrane shrinkage and loss of cell and cell-to-cell contact, and stars indicate the accumulation of material inside cytosolic vesicles. (**B**,**C**) Cell survival was assayed with the MTT method at increasing concentrations of IMI for 24 h (**B**) or 48 h (**C**). *** *p* < 0.001 as compared with control cells. Data are based on three independent experiments.

To verify whether IMI also induces cell death and morphological changes in normal human cells, we tested the effects of IMI on human AG01518 fibroblast cells (Figure 2A–C). Cells were treated with different concentrations of IMI for 24 or 48 h, and we found that IMI induced cytotoxicity above 0.8 mM, as analysed with the MTT assay (Figure 2B,C), which is a substantially higher concentration compared with the D384 cells. Interestingly, we also observed an accumulation of material in a vesicular pattern at concentrations > 0.4 mM (Figure 2A, highlighted with stars).

### 3.3. IMI Exposure Induces ROS Production, Apoptosis, and LMP in Human D384 Astrocytes

**Figure 3:**
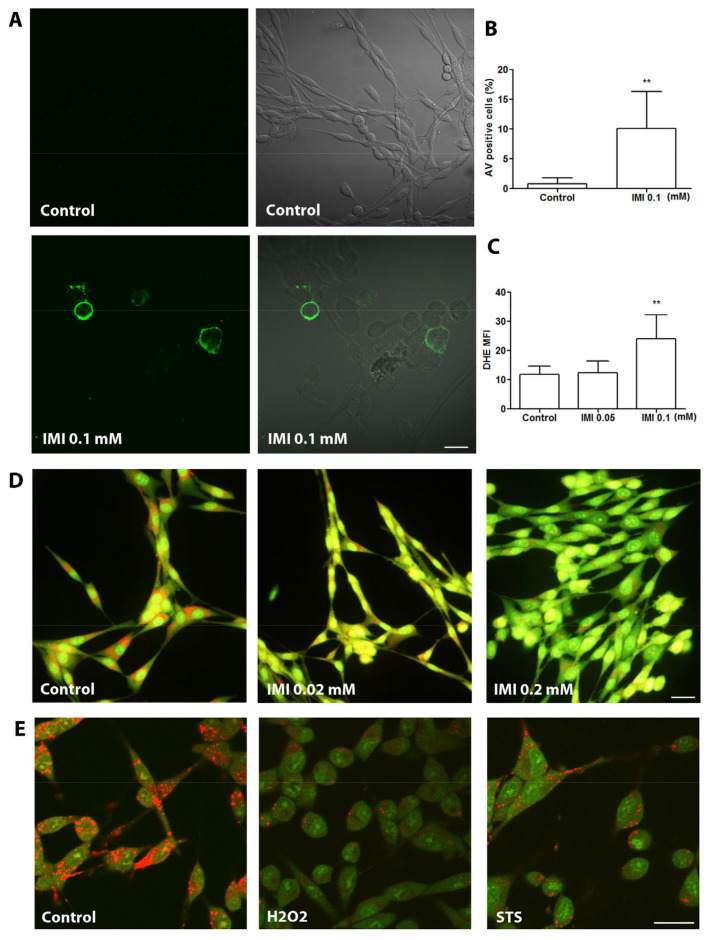
IMI causes apoptosis, ROS production, and LMP of human astrocytoma cell line D384 cells. D384 cells were cultured for 24 h and stained with AV/PI for the detection of apoptosis, stained with DHE as an index for ROS production, or stained with AO for the detection of LMP. (**A**) Representative photographs of AV/PI staining. Bar = 50 μm. (**B**) Quantification of AV positive cells; data are based on three independent experiments; ** *p* < 0.01 as compared with control cells. (**C**) Quantification of DHE fluorescence intensity; data are based on three independent experiments: ** *p* < 0.01 as compared with control cells. (**D**) LMP in control cells and cells exposed to 0.02 or 0.2 mM IMI following AO staining and analysed using fluorescence microscopy. Bar = 50 μm. (**E**) Representative photographs of AO stains for detection of LMP in control cells, and cells treated with 0.1 mM hydrogen peroxide (H_2_O_2_) for 5 h and 0.001 mM staurosporine (STS) for 15 h served as positive controls. Bar = 50 μm.

Since it has been reported that IMI exposure induces apoptosis in several types of cells, we next examined whether exposure to IMI induces apoptotic cell death in D384 cells. Apoptotic cell death, observed with AV staining, was prominent in cultures treated with 0.1mM (Figure 3A), with quantification showing a significant increase in AV-positive cells compared with controls (Figure 3B). Since IMI may impact neurobehavioral performance via oxidative stress and apoptosis-dependent cell death [17,23], we aimed to further evaluate and compare the mechanisms behind the toxicity observed, with a focus on the oxidative stress levels and LMP in the IMI-treated cells. Astrocyte cells were either untreated as controls or treated with 0.05 or 0.1 mM IMI for 24 h. ROS production, observed with DHE staining, was significantly increased in cells treated with 0.1 mM IMI compared with controls (Figure 3C).

LMP, observed with AO uptake, was tested to investigate the cellular mechanisms behind IMI-mediated cytotoxicity in D384 astrocytes. During treatment with 0.02 mM IMI, astrocytes already had an increased LMP observed as reduced red lysosomal granular fluorescence compared with the controls (Figure 3D). Exposure to 0.2 mM IMI clearly pronounced cytosolic green fluorescence, indicating extensively increased LMP (Figure 2D). Pronounced LMP induced by exposure to hydrogen peroxide (H_2_O_2_) or STS served as the positive control (Figure 3E).

### 3.4. IMI Exposure Induces Autophagy Dysfunction in Human D384 Astrocytes 

**Figure 4:**
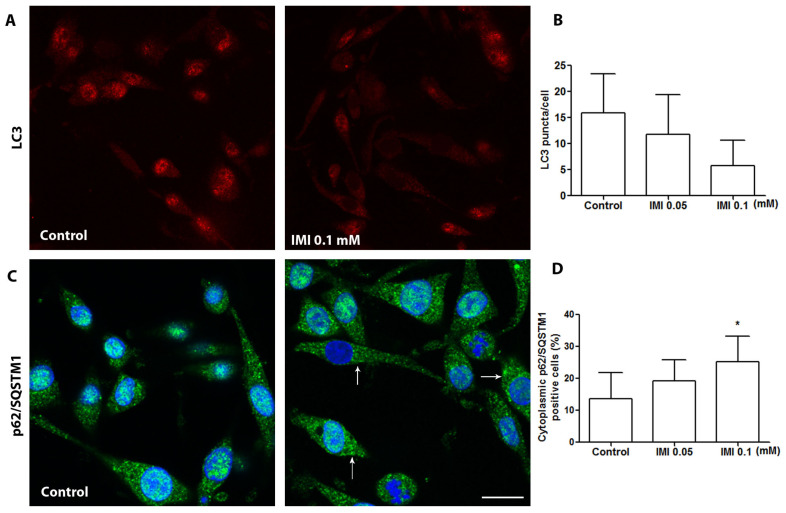
IMI causes autophagy dysfunction. D384 cells were cultured for 24 h in the presence or absence of IMI at different concentrations, as indicated, and then immunostained with LC3 or p62 for fluorescence microscopy. (**A**) Representative photographs of LC3β-immunostained control cells and IMI-exposed cells as indicated. (**B**) Quantification of LC3β puncta in controls and cells treated with IMI. Data are based on three independent experiments. (**C**) Representative photographs of p62/SQSTM1-immunostained control cells and IMI-exposed cells as indicated. Arrows indicate increased cytoplasmic immune granularity of p62/SQSTM1. Bar = 50 μm. (**D**) Quantification of the percentage of cytoplasmic p62/SQSTM1 immuno-intensity. Data are based on two (IMI 0.05 mM) or four (control and IMI 0.1 mM) independent experiments: * *p* < 0.05 as compared with control cells.

Although IMI exposure has been shown to induce an overlap of apoptosis and autophagy in the neurons of honeybees [24,25] and in the *Ctenopharyngodon idellus* kidney cell line [26], there are no data on autophagy status in IMI-exposed astrocytes. Immunocytochemistry was performed to determine the expression and cellular distribution of autophagy markers LC3β and p62/SQSTM1 on D384 astrocytes treated with or without IMI. IMI exposure at 0.1 mM induced a reduction in strong LC3β puncta (Figure 4A,B), as well as increased cytoplasmic levels of p62/SQSTM1 (Figure 4C,D), which indicates dysfunction in autophagic degradation. Moreover, LC3 exhibited a distinctive puncta staining pattern, predominantly located in a nuclear in control cells, but not after IMI treatment (Figure 4A). Interestingly, in some astrocytes, IMI exposure induced cytoplasmic relocation of p62/SQSTM1 in the form of increased cytoplasmic immune granularity of p62/SQSTM1 compared with control astrocytes in cytoplasmytes (Figure 4C,D).

### 3.5. IMI-Induced Autophagy Dysfunction Is Inhibited by 3-Methyladenine but Is Not Affected by Bafilomycin A1 in Human Fibroblast Cells

**Figure 5:**
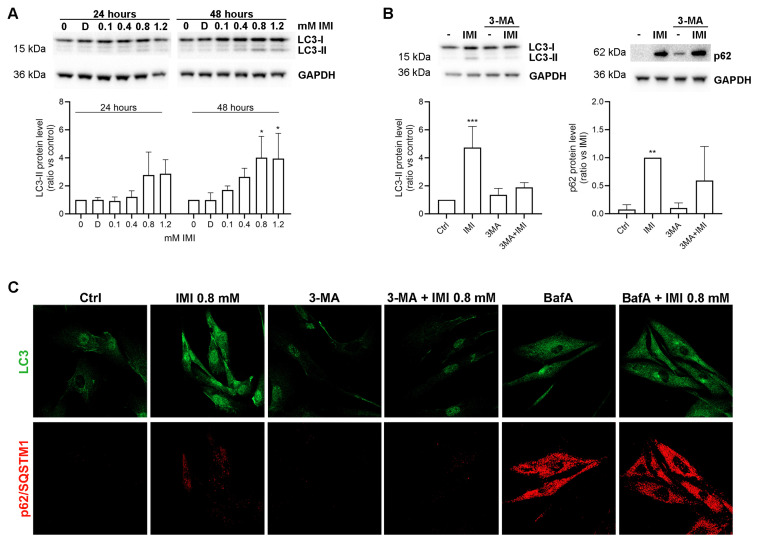
IMI causes autophagy dysfunction in human fibroblast cells. Human fibroblasts were exposed to IMI (0.8 mM for 48 h if not stated otherwise) with or without 3-methyladenine (3-MA, 5 mM) or Bafilomycin A1 (BafA, 25 nM). (**A**) Immunoblot of LC3 at increasing concentrations of IMI, with corresponding densitometric quantifications. D = DMSO control. (**B**) Immunoblotting of LC3 and p62/SQSTM1 with corresponding densitometric quantifications. For p62, protein levels are correlated with IMI due to a lack of signal in controls. (**C**) Representative images of LC3β (green) and p62/SQSTM1 (red) in immunostained cells. Densitometric graphs show mean and SD from three independent experiments: * *p* < 0.05, ** *p* < 0.01, and *** *p* < 0.001.

In human fibroblasts, IMI treatment induced an increase in LC3-II, which was significant first at 0.8 mM for 48 h (Figure 5A). To determine if IMI did in fact cause autophagic dysfunction, we analysed the expression of LC3β and p62/SQSTM1 in the presence of the autophagy inhibitor 3-methyladenine. This inhibitor hinders the formation of the autophagosome [27]. Results show that co-incubating cells with 3-methyladenine reverted the accumulation of LC3-II, as well as p62/SQSTM1, with both immunoblotting (Figure 5B) and immunocytochemistry (Figure 5C). These results suggest that the IMI-induced accumulation of LC3-II occurs downstream of autophagosome formation. Next, we used the lysosomal vacuolar H^+^-ATPase inhibitor Bafilomycin A1, which inhibits both autophagosome–lysosome fusion and lysosomal degradation by reducing lysosomal pH [28]. Contrarily to 3-MA, Bafilomycin A induced a major accumulation of LC3 and p62/SQSTM1 by itself, and the addition of IMI had no apparent effect (Figure 5C), although minor changes might be masked. Taken together, these results indicate that IMI causes dysfunction of autophagy by affecting lysosomal function.

### 3.6. IMI Accumulates in Lysosomes and Reduces Lysosomal Function, Causing Lysophagy and a Compensatory Upregulation of Lysosomes

**Figure 6:**
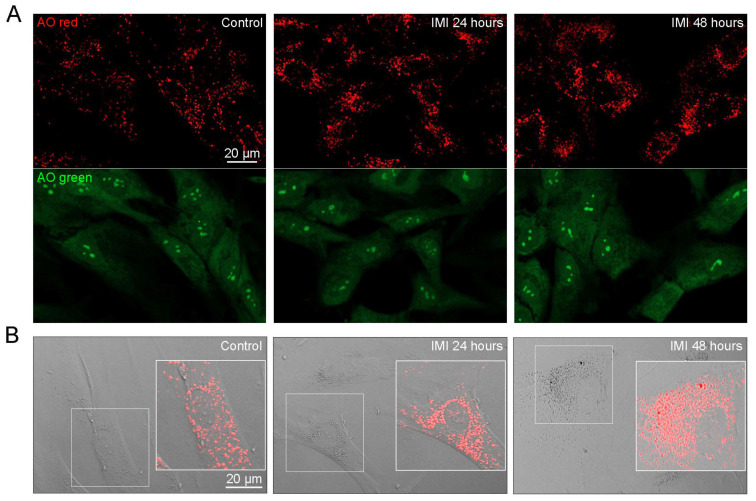

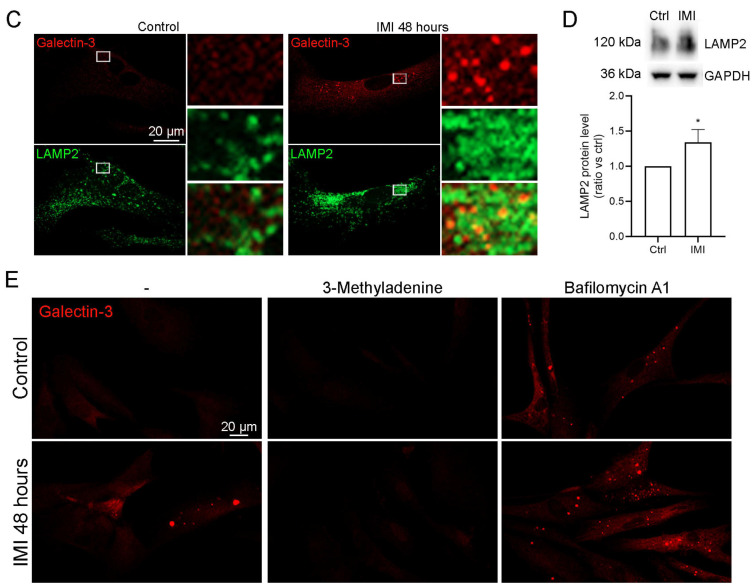
IMI causes lysosomal dysfunction and the upregulation of lysosomal volume. AG01518 fibroblasts were exposed to 0.8 mM IMI for 24 h and 48 h. (**A**) Representative images of acridine orange (AO)-stained cells. (**B**) Combined bright field and red channel of acridine orange fluorescence showing accumulation in lysosomes. (**C**) Representative images of galectin-3 (red) and LAMP2 (green)-immunostained cells, with insets presenting enlarged details. Merged images show co-localisation in yellow. (**D**) Representative immunoblot with corresponding densitometric analysis of LAMP2 after 48 h of IMI exposure. Graph showing mean and SD from three independent experiments; * *p* < 0.05. (**E**) Immunocytochemical images of galectin-3 in cells co-incubated with 0.8 mM IMI and 3-methyladenine (5 mM) or Bafilomycin A1 (25 nM) for 48 h.

To evaluate the effect of IMI on lysosomal function, human fibroblasts were exposed to IMI for 24 and 48 h and stained with AO to visualise lysosomes (red) and lysosomal damage (green). IMI treatment for 48 h slightly augmented both AO red and green fluorescence (Figure 6A). We also observed an increase in the number of AO-positive vesicles after IMI exposure. When combining AO red fluorescence with bright field imaging, it was apparent that the vesicles observed in Figure 2 with accumulated material were indeed lysosomes (Figure 6B). A comparison of 24 h and 48 h confirmed that prolonged exposure to IMI increased both the accumulation of material and the number of lysosomes, suggesting that IMI accumulates within lysosomes and causes a compensatory increase in organelle volume. To further shed light on this mechanism and investigate if lysosomal integrity was compromised, cells were stained for the lysosomal membrane protein LAMP2 and the carbohydrate-binding protein galectin-3. Upon lysosomal damage, galectin-3 binds to exposed glycans in the lysosomal lumen to recruit a specific form of selective autophagy, lysophagy, where the damaged organelle is sequestered and degraded [29,30]. As confirmed in Figure 6C, IMI treatment caused the recruitment of galectin-3, which was localised within vesicles positive for LAMP2. Further, the amount of LAMP2 was greatly increased, which was confirmed with the immunoblot analysis (Figure 6D). Since lysophagy is dependent on the formation of a phagophore to sequester the damaged lysosome, we tested whether inhibition of autophagosome formation using 3-methyladenine had any effect. Indeed, 3-methyladenine decreased the recruitment of galectin-3 (Figure 6E). Surprisingly, we also found that Bafilomycin A1 caused galectin-3 puncta by itself, which was further enhanced when co-incubating cells with IMI (Figure 6E). Although we did not observe that Bafilomycin A1 induces lysosomal damage when incubating for a shorter time [22], the induction of LMP is a known feature of Bafilomycin A1 [31,32]. Together, these results suggest that IMI accumulates in the lysosomes, causing lysosomal leakage and reduced lysosomal function, which induces activation of lysophagy and a compensatory increase in the number of lysosomes and lysosomal proteins.

## 4. Discussion

This investigation aimed to assess the toxic effects of IMI on human astrocytes and fibroblasts. This study was motivated by a clinical case in which a patient developed Bell’s palsy as a suspected response to chronic exposure to plant protection products containing IMI. In this study, we showed that IMI exposure induces cell death in both astrocytes and human fibroblasts, associated with lysosomal dysfunction, ROS production, and autophagy dysfunction.

In addition to individual suicidal case reports, observational studies from Sri Lanka [33] and Thailand [34] observed that IMI exposure induces mild toxicity in humans. Human exposure to IMI can occur through a variety of pathways, including inhalation, dermal contact, and dietary intake. Studies have shown that imidacloprid residues can persist in a variety of food products, raising concerns about its potential impacts on consumers [35]. A low mortality rate was observed in the Thai study (3.1%) [34], whereas no deaths were recorded in the Sri Lankan study [33]. Common toxic effects were associated with gastrointestinal, cardiovascular, respiratory (dyspnoea and respiratory depression), and CNS (coma) effects [33,34].

It has been shown that IMI is metabolised to its more toxic metabolite imidacloprid-olefin in animals and plants. The olefinic metabolite has been observed to be more toxic/effective than the parent compound [36,37]. Notably, chronic exposure to IMI often leads to “unexplainable” cumulative and delayed effects, which may be due to secondary mechanisms of action of IMI and its toxic metabolite [38]. Herein, our case patient developed Bell’s palsy and experienced mild gastrointestinal effects, which may relate to chronic occupational exposure to an IMI-containing plant protection product and the toxic effects of IMI metabolites. 

Due to the previous clinical observations of neurological effects, and this specific case of Bell’s palsy, we aimed to investigate the effects of IMI on astrocytes, a CNS-specific cell type. Previously, IMI demonstrated toxicity towards human neuronal SH-SY5Y cells, affecting nAChR signalling [10,12]. Herein, we further demonstrated that IMI induces cell death in a non-neuronal glial cell type—D384 astrocytes. In the current study, assessment of DNA quantities, as a reflection of viable D384 astrocytes, demonstrated a reduction in cellular DNA quantity at 0.1 mM IMI with a significant decrease at 0.8 mM, with the observed dose-dependent relationship between the number of necrotic cells and IMI treatment, with a significant increase at 0.8 mM IMI after 24 h. Similarly, a dose-dependent relationship was observed between IMI treatment and the induction of DNA damage, with significant DNA damage at 0.5 mM IMI [10], with the suggestion that low expression of heat shock proteins (HSPs), specifically HSP27 as an anti-apoptosis factor, may contribute to the increase in cell mortality. These results, with previous findings, may suggest a relationship between IMI toxicity and the expression of stress proteins like HSPs.

Whilst the mechanisms of action underlying IMI toxicity in human cells remain unclear, here, we show that apoptosis was present alongside the involvement of ROS production and LMP. To the best of our knowledge, this is the first study to assess both ROS production and LMP in a human CNS-based cell model of IMI exposure. Herein, we show an increase in ROS production via significantly increased DHE-positive staining in IMI-treated astrocytes. In agreement with our findings, increases in oxidative stress and ROS production in response to IMI exposure have been observed in non-CNS cell lines. In the HepG2 hepatic cell line, a significant decrease in the reduced/oxidised glutathione ratio, indicative of oxidative stress, was observed after 0.5 mM IMI treatment over 24 h [39]. In the colorectal HT-29 cell line, increased oxidative stress and ROS were observed in experiments of both pure IMI and commercial formulations of IMI [16]. In the WPM-Y.1 prostate epithelial cell line, significant increases in the enzymatic activities of catalase, glutathione peroxidase, and glutathione reductase were also observed when comparing IMI-treated and control cells [9]. Furthermore, we observed a time-dependent accumulation of IMI inside lysosomes of human fibroblasts. The accumulation caused lysosomal damage and inhibited autophagy, causing a compensatory upregulation of lysosomes. Lysosomal dysfunction due to a build-up of undegradable material is a known contributing cause of several neurodegenerative diseases [40,41]. In these disorders, lysosomes fail to degrade misfolded protein oligomers (e.g., Aβ in Alzheimer’s disease and α-synuclein in Parkinson’s disease), which results in the accumulation of neurotoxic aggregates inside the lysosomal lumen. The cumulative build-up inhibits normal lysosomal function and eventually induces lysosomal damage [42]. The release of cathepsins and other lysosomal hydrolases induces inflammatory processes, eventually leading to cell death and neurodegeneration [43,44].

IMI-induced autophagy has not previously been investigated in human cell models. However, several animal models within the context of environmental contamination have highlighted autophagy dysfunction as a result of IMI exposure. Studies investigating the honeybee (*Apis mellifera*) and the response to IMI exposure found increased apoptosis with autophagic features using transmission electron microscopy in brain tissues [25], midgut epithelium [24], and fish kidney cells [26]. In Pacific white shrimp (*Litopenaeus vannamei*), the inhibition of autophagic pathways was suggested as the toxic mechanism of IMI [45]. Herein, we show that IMI induces autophagy dysfunction with altered LC3β and p62/SQSTM1 expressions. Our study, together with these animal studies, indicates that IMI has a clear cytotoxic effect by inducing apoptosis in a variety of cell types and that autophagy dysfunction may be involved. However, more studies are required, specifically in human models, to expand the knowledge of the potential contribution of autophagy in IMI-exposed cells.

## 5. Conclusions

Our study is the first to investigate the cytotoxic effects of IMI on the human D384 astrocyte cell line. IMI was observed to induce astrocyte apoptosis and necrosis with associated ROS production, LMP, and autophagy dysfunction. Functional studies on normal fibroblasts revealed that the autophagic dysfunction was caused by an accumulation of IMI inside lysosomes, leading to lysosomal damage, activation of lysophagy, and a compensatory increase in lysosomes. The observed IMI-induced effects on the CNS-specific cell type provide a possible link between IMI cytotoxicity and neurological complications observed clinically in patients exposed to this neonicotinoid insecticide. More studies are required to elucidate the mechanisms of action of IMI in heterogeneous cell models and the reaction in vivo. In addition, the environmental impact of IMI contamination needs to be further assessed in relation to possible detrimental effects on human health and the environment.

## Data Availability

The authors confirm that the data supporting the findings of this study are available within the article. Experimental data that support the findings of this study are also available from the corresponding author upon reasonable request.

## References

[1] Hladik M.L., Main A.R., Goulson D. (2018). Environmental Risks and Challenges Associated with Neonicotinoid Insecticides. Environ. Sci. Technol..

[2] Pang S., Lin Z., Zhang Y., Zhang W., Alansary N., Mishra S., Bhatt P., Chen S. (2020). Insights into the Toxicity and Degradation Mechanisms of Imidacloprid Via Physicochemical and Microbial Approaches. Toxics.

[3] Herbertsson L., Jonsson O., Kreuger J., Smith H.G., Rundlöf M. (2021). Scientific Note: Imidacloprid Found in Wild Plants Downstream Permanent Greenhouses in Sweden. Apidologie.

[4] Boye K., Boström G., Jonsson O., Gönczi M., Löfkvist K., Kreuger J. (2022). Greenhouse Production Contributes to Pesticide Occurrences in Swedish Streams. Sci. Total Environ..

[5] Boye K., Lindström B., Boström G., Kreuger J. (2019). Long-term Data from the Swedish National Environmental Monitoring Program of Pesticides in Surface Waters. J. Environ. Qual..

[6] Distefano G.G., Zangrando R., Basso M., Panzarin L., Gambaro A., Volpi Ghirardini A., Picone M. (2022). The Ubiquity of Neonicotinoid Contamination: Residues in Seabirds with Different Trophic Habits. Environ. Res..

[7] Costa C., Silvari V., Melchini A., Catania S., Heffron J.J., Trovato A., de Pasquale R. (2009). Genotoxicity of Imidacloprid in Relation to Metabolic Activation and Composition of the Commercial Product. Mutat. Res. Genet. Toxicol. Environ. Mutagen..

[8] Feng S., Kong Z., Wang X., Peng P., Zeng E.Y. (2005). Assessing the Genotoxicity of Imidacloprid and RH-5849 in Human Peripheral Blood Lymphocytes in vitro with Comet Assay and Cytogenetic Tests. Ecotoxicol. Environ. Saf..

[9] Abdel-Halim K.Y., Osman S.R. (2020). Cytotoxicity and Oxidative Stress Responses of Imidacloprid and Glyphosate in Human Prostate Epithelial WPM-Y.1 Cell Line. J. Toxicol..

[10] Skandrani D., Gaubin Y., Beau B., Murat J.C., Vincent C., Croute F. (2006). Effect of Selected Insecticides on Growth Rate and Stress Protein Expression in Cultured Human A549 and SH-SY5Y Cells. Toxicol. Vitr..

[11] Şenyildiz M., Kilinc A., Ozden S. (2018). Investigation of the Genotoxic and Cytotoxic Effects of Widely Used Neonicotinoid Insecticides in HepG2 and SH-SY5Y Cells. Toxicol. Ind. Health.

[12] Loser D., Hinojosa M.G., Blum J., Schaefer J., Brüll M., Johansson Y., Suciu I., Grillberger K., Danker T., Möller C. (2021). Functional Alterations by a Subgroup of Neonicotinoid Pesticides in Human Dopaminergic Neurons. Arch. Toxikol..

[13] Nakayama A., Yoshida M., Kagawa N., Nagao T. (2019). The Neonicotinoids Acetamiprid and Imidacloprid Impair Neurogenesis and Alter the Microglial Profile in the Hippocampal Dentate Gyrus of Mouse Neonates. J. Appl. Toxicol..

[14] Reinwald H., Alvincz J., Salinas G., Schäfers C., Hollert H., Eilebrecht S. (2022). Toxicogenomic Profiling after Sublethal Exposure to Nerve- and Muscle-Targeting Insecticides Reveals Cardiac and Neuronal Developmental Effects in Zebrafish Embryos. Chemosphere.

[15] Janner D.E., Gomes N.S., Poetini M.R., Poleto K.H., Musachio E.A.S., de Almeida F.P., de Matos Amador E.C., Reginaldo J.C., Ramborger B.P., Roehrs R. (2021). Oxidative stress and decreased dopamine levels induced by imidacloprid exposure cause behavioral changes in a neurodevelopmental disorder model in Drosophila melanogaster. Neurotoxicology.

[16] Baysal M., Atlı-Eklioğlu Ö. (2021). Comparison of the Toxicity of Pure Compounds and Commercial Formulations of Imidacloprid and Acetamiprid on HT-29 Cells: Single and Mixture Exposure. Food Chem. Toxicol..

[17] Silva A.M., Martins-Gomes C., Ferreira S.S., Souto E.B., Andreani T. (2022). Molecular Physicochemical Properties of Selected Pesticides as Predictive Factors for Oxidative Stress and Apoptosis-Dependent Cell Death in Caco-2 and HepG2 Cells. Int. J. Mol. Sci..

[18] Quent V.M.C., Loessner D., Friis T., Reichert J.C., Hutmacher D.W. (2010). Discrepancies between Metabolic Activity and DNA Content as Tool to Assess Cell Proliferation in Cancer Research. J. Cell Mol. Med..

[19] Yuan X.M., Li W., Olsson A.G., Brunk U.T. (1997). The Toxicity to Macrophages of Oxidized Low-Density Lipoprotein Is Mediated through Lysosomal Damage. Atherosclerosis.

[20] Ladoire S., Chaba K., Martins I., Sukkurwala A.Q., Adjemian S., Michaud M., Poirier-Colame V., Andreiuolo F., Galluzzi L., White E. (2012). Immunohistochemical Detection of Cytoplasmic LC3 Puncta in Human Cancer Specimens. Autophagy.

[21] Schneider C.A., Rasband W.S., Eliceiri K.W. (2012). NIH Image to ImageJ: 25 Years of Image Analysis. Nat. Methods.

[22] Eriksson I., Wäster P., Öllinger K. (2020). Restoration of lysosomal function after damage is accompanied by recycling of lysosomal membrane proteins. Cell Death Dis..

[23] Abd-Elhakim Y.M., Mohammed H.H., Mohamed W.A.M. (2018). Imidacloprid Impacts on Neurobehavioral Performance, Oxidative Stress, and Apoptotic Events in the Brain of Adolescent and Adult Rats. J. Agric. Food Chem..

[24] Carneiro L.S., Martinez L.C., de Oliveira A.H., Cossolin J.F.S., de Resende M.T.C.S., Gonçalves W.G., Medeiros-Santana L., Serrão J.E. (2022). Acute Oral Exposure to Imidacloprid Induces Apoptosis and Autophagy in the Midgut of Honey Bee Apis Mellifera Workers. Sci. Total Environ..

[25] Wu Y.Y., Zhou T., Wang Q., Dai P.L., Xu S.F., Jia H.R., Wang X. (2015). Programmed Cell Death in the Honey Bee (*Apis mellifera*) (Hymenoptera: Apidae) Worker Brain Induced by Imidacloprid. J. Econ. Entomol..

[26] Li X., Yao Y., Wang J., Shen Z., Jiang Z., Xu S. (2022). Eucalyptol Relieves Imidacloprid-Induced Autophagy through the MiR-451/Cab39/AMPK Axis in Ctenopharyngodon Idellus Kidney Cells. Aquat. Toxicol..

[27] Seglen P.O., Gordon P.B. (1982). 3-Methyladenine: Specific inhibitor of autophagic/lysosomal protein degradation in isolated rat hepatocytes. Proc. Natl. Acad. Sci. USA.

[28] Mauvezin C., Neufeld T.P. (2015). Bafilomycin A1 disrupts autophagic flux by inhibiting both V-ATPase-dependent acidification and Ca-P60A/SERCA-dependent autophagosome-lysosome fusion. Autophagy.

[29] Chauhan S., Kumar S., Jain A., Ponpuak M., Mudd M.H., Kimura T., Choi S.W., Peters R., Mandell M., Bruun J.A. (2016). TRIMs and galectins globally cooperate and TRIM16 and galectin-3 co-direct autophagy in endomembrane damage homeostasis. Dev. Cell.

[30] Maejima I., Takahashi A., Omori H., Kimura T., Takabatake Y., Saitoh T., Yamamoto A., Hamasaki M., Noda T., Isaka Y. (2013). Autophagy sequesters damaged lysosomes to control lysosomal biogenesis and kidney injury. EMBO J..

[31] Li Y., Sun Y., Jing L., Wang J., Yan Y., Feng Y., Zhang Y., Liu Z., Ma L., Diao A. (2017). Lysosome Inhibitors Enhance the Chemotherapeutic Activity of Doxorubicin in HepG2 Cells. Chemotherapy.

[32] Shaikh S., Nandy S.K., Cantí C., Lavandero S. (2019). Bafilomycin-A1 and ML9 Exert Different Lysosomal Actions to Induce Cell Death. Curr. Mol. Pharmacol..

[33] Mohamed F., Gawarammana I., Robertson T.A., Roberts M.S., Palangasinghe C., Zawahir S., Jayamanne S., Kandasamy J., Eddleston M., Buckley N.A. (2009). Acute Human Self-Poisoning with Imidacloprid Compound: A Neonicotinoid Insecticide. PLoS ONE.

[34] Sriapha C., Trakulsrichai S., Tongpoo A., Pradoo A., Rittilert P., Wananukul W. (2020). Acute Imidacloprid Poisoning in Thailand. Ther. Clin. Risk Manag..

[35] Bonmatin J.M., Giorio C., Girolami V., Goulson D., Kreutzweiser D.P., Krupke C., Liess M., Long E., Marzaro M., Mitchell E.A. (2015). Environ-mental fate and exposure neonicotinoids and fipronil. Environ. Sci. Pollut. Res. Int..

[36] Suchail S., Guez D., Belzunces L.P. (2001). Discrepancy between Acute and Chronic Toxicity Induced by Imidacloprid and Its Metabolites in Apis Mellifera. Environ. Toxicol. Chem..

[37] Seifrtova M., Halesova T., Sulcova K., Riddellova K., Erban T. (2017). Distributions of Imidacloprid, Imidacloprid-Olefin and Imidacloprid-Urea in Green Plant Tissues and Roots of Rapeseed (*Brassica napus*) from Artificially Contaminated Potting Soil. Pest. Manag. Sci..

[38] Erban T., Sopko B., Talacko P., Harant K., Kadlikova K., Halesova T., Riddellova K., Pekas A. (2019). Chronic Exposure of Bumblebees to Neonicotinoid Imidacloprid Suppresses the Entire Mevalonate Pathway and Fatty Acid Synthesis. J. Proteomics.

[39] Guimarães A.R.D.J.S., Bizerra P.F.V., Miranda C.A., Mingatto F.E. (2022). Effects of Imidacloprid on Viability and Increase of Reactive Oxygen and Nitrogen Species in HepG2 Cell Line. Toxicol. Mech. Methods.

[40] Wolfe D.M., Lee J.H., Kumar A., Lee S., Orenstein S.J., Nixon R.A. (2013). Autophagy failure in Alzheimer’s disease and the role of defective lysosomal acidification. Eur. J. Neurosci..

[41] Vidyadhara D.J., Lee J.E., Chandra S.S. (2019). Role of the endolysosomal system in Parkinson’s disease. J. Neurochem..

[42] Flavin W.P., Bousset L., Green Z.C., Chu Y., Skarpathiotis S., Chaney M.J., Kordower J.H., Melki R., Campbell E.M. (2017). Endocytic vesicle rupture is a conserved mechanism of cellular invasion by amyloid proteins. Acta. Neuropathol..

[43] Dehay B., Bove J., Rodriguez-Muela N., Perier C., Recasens A., Boya P., Vila M. (2010). Pathogenic lysosomal depletion in Parkinson’s disease. J. Neurosci..

[44] Hook G., Hook V., Kindy M. (2011). The cysteine protease inhibitor, E64d, reduces brain amyloid-beta and improves memory deficits in Alzheimer’s disease animal models by inhibiting cathepsin B, but not BACE1, beta-secretase activity. J. Alzheimers. Dis..

[45] Fu Z., Han F., Huang K., Zhang J., Qin J.G., Chen L., Li E. (2022). Impact of Imidacloprid Exposure on the Biochemical Responses, Transcriptome, Gut Microbiota and Growth Performance of the Pacific White Shrimp Litopenaeus Vannamei. J. Hazard Mater..

